# Impact of neurocognitive deficits on patient–proxy agreement regarding health-related quality of life in low-grade glioma patients

**DOI:** 10.1007/s11136-016-1426-z

**Published:** 2016-10-15

**Authors:** Divine E. Ediebah, Jaap C. Reijneveld, Martin J. B. Taphoorn, Corneel Coens, Efstathios Zikos, Neil K. Aaronson, Jan J. Heimans, Andrew Bottomley, Martin Klein, on behalf of the EORTC Quality of Life Department and Patient Reported Outcome and Behavioral Evidence (PROBE)

**Affiliations:** 1 Quality of Life Department, European Organisation for Research and Treatment of Cancer (EORTC), Brussels, Belgium; 2 Department of Neurology, VU University Medical Center, Amsterdam, The Netherlands; 3 Department of Medical Psychology – B7D349, VU University Medical Center, Van der Boechorststraat 7, 1081 BT Amsterdam, The Netherlands; 4 Department of Neurology, Medical Center Haaglanden, The Hague, The Netherlands; 5 Division of Psychosocial Research and Epidemiology, The Netherlands Cancer Institute, Amsterdam, The Netherlands

**Keywords:** Brain tumor, Health-related quality of life, Proxy ratings, Neurocognitive deficits

## Abstract

**Purpose:**

Clinical trials in glioma patients with neurocognitive deficits face challenges due to lacking or unreliable patient self-reports on their health-related quality of life (HRQOL). Patient–proxy data could help solve this issue. We determined whether patient–proxy concordance levels were affected by patients’ neurocognitive functioning.

**Methods:**

Patient and patient-by-proxy HRQOL ratings were assessed via SF-36 and EORTC QLQ-BN20, respectively, in 246 patients. Data on neurocognitive functioning were collected on a subgroup of 195 patients. Patient–proxy agreement was measured using the Bland–Altman limit of agreement, the mean difference, the concordance correlation coefficient (CCC), and the percentage difference (PD, ±0, 5, or 10 points). We defined patients to be cognitively impaired (*n* = 66) or cognitively intact (*n* = 129) based on their neurocognitive performance.

**Results:**

Patients rated their physical function and general health to be better than their proxies did, while at the same time, patients reported more visual disorders, communication deficits, itchy skin, and problems with bladder control. The cognitively impaired subgroup reported poorer physical functioning, more visual disorders, headaches, itchy skin, and issues with bladder control. In the cognitively intact group, no statistical significant differences were observed between patients and proxies. Not surprisingly, Bland–Altman plots revealed a high agreement between the patient and patient-by-proxy rating in all HRQOL domains ranging from 95 to 99 %. The CCC was fairly high in all HRQOL domains (0.37–0.80), and the percentage of perfect agreement (PD ± 0) ranged from 8.5 to 76.8 %. In the cognitively impaired patients, the mean difference between patients and proxies was overall larger, and accordingly, agreement based on Bland–Altman plots was lower.

**Conclusions:**

The level of agreement between patient and patient-by-proxy ratings of low-grade glioma patients’ HRQOL is generally high. However, patient–proxy agreement is lower in patients with neurocognitive deficits than in patients without neurocognitive deficits.

**Electronic supplementary material:**

The online version of this article (doi:10.1007/s11136-016-1426-z) contains supplementary material, which is available to authorized users.

## Introduction

Health-related quality of life (HRQOL) has become an important secondary outcome measure in clinical trials of glioma patients [1, 2] with all European Organization for Research and Treatment of Cancer (EORTC) brain tumor clinical trials and most trials by other cancer groups now incorporating HRQOL. HRQOL is an important complement to conventional outcome parameters such as time to tumor progression, overall and progression-free survival, and radiological response might be inadequate or less relevant for meaningful evaluation of this type of treatment [3]. HRQOL in glioma patients is influenced by both tumor- and treatment-related factors [4, 5] with seizures and antiepileptic drugs [6], fatigue [7], anxiety and depression [8], and neurocognitive deficits [9, 10] affecting HRQOL in particular. Apart from negatively affecting HRQOL, neurocognitive deficits may also hamper adequate patient self-reports, as patients’ neurocognitive deficits may render HRQOL patient-reported outcomes through questionnaires unreliable [11]. Exclusion of these patients at the lower end of the neurocognitive spectrum from analyses obviously introduces undesirable bias in the evaluation of patients’ HRQOL during experimental treatments. Moreover, cognitively impaired patients may be less compliant regarding questionnaire-based HRQOL monitoring, thereby introducing another source of bias.

The incorporation of HRQOL estimates of the partner or another close relative or friend (denominated as ‘proxy’) might solve this problem to a large extent. Previous reports indicate that high-grade glioma patient- and proxy-reported HRQOL have a high level of concordance as long as the patient shows no signs of decline in neurocognitive function [12, 13], but differences, particularly in mood-related issues, increase when neurocognitive functioning decreases [13, 14]. While HRQOL is by definition subjective, and it is typically measured with self-reports, it has been suggested that substituting proxy ratings when a patient’s self-report is absent or unreliable should be considered [15]. When differences between patient- and proxy-reported HRQOL ratings develop in the course of the disease (presumably at the time, decline in neurocognitive function becomes an issue), proxy-reported instead of patient-reported HRQOL ratings might be regarded as the most reliable source of information on patients’ HRQOL.

Previous studies reported that low observed correlations between patient- and patient-by-proxy-reported outcomes might be explained by methodological weaknesses such as small sample size, suboptimal reliability, and score variability [13, 14]. This was supported by Bland and Altman, who stated that a single measure such as a correlation coefficient may not be sufficient to summarize agreement adequately [16, 17]. In the present study, by using a wide range of statistical measures of agreement, we investigated patient–proxy HRQOL agreement in a large sample of low-grade glioma (LGG) patients, both with intact and with impaired neurocognitive functioning. The cohort used in this analysis is unique because of the extensive neurocognitive test battery incorporated.

The aim of the present study is to investigate the agreement between patient and patient-by-proxy ratings of HRQOL and to investigate whether the level of neurocognitive functioning influences the level of patient–proxy concordance. We hypothesized that (1) concordance levels are relatively high on mental and physical functioning in cognitively intact patients, and (2) there is a decrease in mental functioning in cognitively impaired patients, with proxies being more negative on patients’ HRQOL.

## Patients and methods

For this cross-sectional study, we recruited low-grade glioma (LGG) patients who were disease-free for at least one year following diagnosis and primary treatment, and their proxies. Patients were recruited from neurosurgical centers throughout the Netherlands between February, 1997 and January, 2000. Eligibility was checked with the general practitioner and by a case-note review. Low-grade glioma was classified histologically as astrocytoma, oligodendroglioma, or oligoastrocytoma. Patients were excluded if they used corticosteroids (because use of corticosteroids might indicate non-stable disease), did not have a basic proficiency in the Dutch language, or were unable to communicate adequately. All patients provided written informed consent to be involved on the study, and ethics approvals of the study protocol were obtained from the medical ethics committees of the institutions. The details of the study conduct and clinical outcome have been reported elsewhere [18].

### Health-related quality-of-life assessments

HRQOL was assessed using the *MOS SF*-*36 Short*-*Form Health Survey* (SF-36) [19, 20] in conjunction with the European Organization for Research and Treatment of Cancer Brain Cancer module (QLQ-BN20) [21] to assess additional health problems associated specifically with glioma and its treatment. The MOS SF-36 is a self-report questionnaire developed in the USA as a part of a large, national study of the effect of various forms of health care delivery on patients’ health status and quality of life [22]. It is composed of 36 items, organized into eight multi-item scales assessing: (1) physical functioning; (2) bodily pain; (3) role limitations due to physical health problems; (4) role limitations due to personal or emotional problems; (5) emotional well-being; (6) social functioning; (7) energy/fatigue; and (8) general health perceptions. Summary component scores for physical health (PCS) and mental health (MCS) were also calculated. Higher scores indicate better health. The questionnaire has excellent reliability and validity when employed with diverse patient populations [23, 24]. The SF-36 also has exhibited good validity and reliability (Cronbach’s alpha = 0.84) when employed among Dutch cancer patients [25].

The QLQ-BN20, where higher scores indicate *more* symptoms, is composed of 20 items, organized into five subscales assessing future uncertainty, visual disorder, motor dysfunction, communication deficit, and emotional distress. The remaining seven items assess other disease symptoms, and side effects of treatment found to be prevalent among patients with brain tumors, including headaches, seizures, drowsiness, hair loss, itching, weakness in the legs, and lack of bladder control. The QLQ-BN20 has robust psychometric properties that result from rigorous testing and the development of their use in several international clinical cancer trials [26]. The SF-36 and QLQ-BN20 were completed by (1) the patient; (2) the partner, providing a ‘proxy’ rating of the patients’ HRQOL, i.e., patient-by-proxy. Based on the objective outlined in the introduction, we will limit our analysis of agreement to SF-36 and QLQ-BN20 questionnaires completed by the patient and the partner as a proxy.

### Neurocognitive assessments

Neurocognitive functioning refers to an individual’s ability to perceive, store, retrieve, and use sensory and perceptual information from the environment and past experience, and to such mental activities as plans and strategies [18]. A disability score was calculated for every patient; neurocognitive test scores were converted to z-scores, with the mean scores of the healthy controls as a reference. The lower 5th percentile of the healthy controls was used as a cut-off score for cognitive disability [27]. To calculate an overall disability score for every patient, we counted the number of tests on which the patient scored below this cut-off. Application of this algorithm to our data showed that a glioma patient was judged to have a neurocognitive disability if he or she had deviant scores for at least 4 of the 20 tests. Only tests for which healthy controls could be individually matched with LGG patients for age, sex, and educational level were used for this analysis. Unlike, for instance, research concerning patients with Alzheimer’s disease (AD), there is no consensus on what represents a ‘true’ drop in neurocognitive functioning in brain tumor patients. By applying this strict, clinically based cut-off, we assume that patients with neurocognitive disability will experience limitations in their daily life functioning. Detailed information about the standard tests used to assess cognitive status is shown in Table 1.

**Table 1:** Neuropsychological tests and corresponding cognitive domains

*Intelligence*	
Dutch adult reading test [30]	Estimates premorbid intellectual functioning on the basis of verbal ability
*Perception and psychomotor speed*	
Line bisection test [31]	Measures unilateral neglect, which is usually a sequel of massive right hemisphere lesions
Facial recognition test [31]	Detects impairment in the discrimination of faces, a disorder associated with right hemisphere lesions
Judgment of line orientation test [31]	A test of visuospatial judgment, also detects right hemisphere dysfunction
Letter-digit substitution test (LDST) [31]	Measures psychomotor speed that is relatively unaffected by a decline in intellectual ability
*Memory*	
Visual verbal learning test (VVLT) [31]	Examines several aspects of verbal learning, organization, and memory
Working memory task (WMT) [31]	Measures the speed of memory processes
*Attention and executive function*	
Stroop color-word test (SCWT) [31]	Examines attention, mental speed, and mental control
Categoric word fluency task [31]	Measures the speed and flexibility of verbal thought processes
Concept shifting test (CST) [32]	Measures attention, visual searching, mental-processing speed, and the ability to mentally control simultaneous stimulus patterns

### Functional/performance status

Patient’s *performance status* was assessed with the Karnofsky performance status scale (KPS). [28, 29] This physician-rated scale is used widely as an outcome measure in cancer clinical studies.

The capacity to carry out *activities of daily living* (ADL) was assessed with the Barthel Index [30]. This index consists of 10 items assessing: continence of bowels and bladder, grooming, toilet use, feeding, transfer, mobility, dressing, climbing stairs, and bathing. The items are ordered in ascending degree of difficulty. It has proven to be a reliable and valid instrument for assessing disability in basic activities of daily living (ADL) and mobility and has been used primarily with stroke patients.

*Neurological functioning* was rated with the neurological functional status scale developed by Order et al. [31]. Scores range from 1 to 4, with high scores for strong neurological function.

### Statistical analysis

The raw scores from both the SF-36 and QLQ-BN20 were transformed to a linear scale that ranged from 0 to 100, in which a higher score represents a higher level of functioning or a worse level of symptoms [32, 33]. The following techniques were used to measure the agreement between the patient and the patient-by-proxy HRQOL scores. The mean differences (patient-by-proxy minus patient) on the SF-36 and QLQ-BN20 were summarized as means and SD. Wilcoxon signed-rank test was used to test the differences between patient and patient-by-proxy scores. The proportion of subjects whose difference between patient and patient-by-proxy scores was within ±0, 5 and 10 units [34, 35] was summarized. A difference within ±0 was considered as perfect agreement. Bland–Altman limits of agreement (LA) [16, 36–39] were created to measure the agreement between patient and patient-by-proxy scores and to demonstrate the extent of rater disagreement across the range of a scale (i.e., to evaluate the magnitude of disagreement, the identification of outliers, and the observation of any bias) for each method of measurement. The Bland–Altman method depicts the mean difference between two methods of measurement (the ‘bias’), and 95 % limits of agreement (prediction interval) as the mean difference (2 SD) [or more precisely (1.96 SD)]. It is the pattern of the data points that identify agreement, types of bias and outliers. It is expected that the 95 % limits include 95 % of the data point differences between the two measurements, i.e., about 95 % of the points should lie with the interval. Lin’s concordance correlation coefficient (CCC) [40, 41] was also computed for patient and patient-by-proxy scores.

To investigate the effect of neurocognitive function on the patient–proxy agreement, we defined patients as being cognitively impaired (*n* = 66) or cognitively intact (*n* = 129) based on their neuropsychological performance. We then assessed the level of agreement via LA for PCS and MCS and mean difference for both the SF-36 and QLQ-BN20 scales.

## Results

In this nationwide study [18] into HRQOL and neurocognitive functioning in low-grade glioma patients, we recruited 281 adult patients with supratentorial low-grade gliomas who were asked to complete the SF-36 and QLQ-BN20 scales. Their proxies were asked to complete the questionnaire on the patient’s SF-36 and QLQ-BN20; 35 of the 281 glioma patients (12.5 %) had no patient-by-proxy assessment and were thus excluded from the analyses. Forty-four of the 239 glioma patients (18 %) who met the inclusion criteria and who were asked to participate in the neurocognitive part of the study declined to participate; the main reasons being that participation was too burdensome, or that they were reluctant to be confronted with what they believed to be a cured illness. Neurocognitive data were available for 195 patients (87.5 %), of whom 104 (53 %) had received radiotherapy 1–22 years previously. Ninety-three percent of glioma patients were tested at home; the remainder were tested in the hospital. The clinical characteristics of the LGG patients have been described previously [18]. Briefly, Table 2 shows the sociodemographic and clinical characteristics of the LGG patients. The mean age was 42 years, with the majority being men (62 %). The histological diagnosis was astrocytoma (71 %), oligodendroglioma (22 %), and oligoastrocytoma (7 %). Patients’ performance status (KPS), their capacity to carry out activities of daily living (Barthel), and neurological functioning (Order) reached near-optimal levels.

**Table 2:** Sociodemographic and clinical characteristics

Characteristics	LGG patients (*n* = 195)
Age in years, mean (SD)	40.79 (11.62)
Male sex, *n* (%)	120 (61.54 %)
Level of education, mean (SD)	4.16 (2.09)
Radiotherapy, *n* (%)	
Yes	104 (53.33 %)
No	91 (46.67 %)
Premorbid intelligence, mean (SD)	
Dutch adult reading test	99.94 (10.78)
Histological diagnosis, *n* (%)	
Astrocytoma	139 (71.28 %)
Oligodendroglioma	43 (22.05 %)
Oligoastrocytoma	13 (6.67 %)
Tumor lateralization, *n* (%)	
Left-sided	97 (49.74 %)
Right-sided	91 (46.67 %)
Bilateral	7 (3.59 %)
Neurosurgical intervention, *n* (%)	
Biopsy	84 (43.08 %)
Resection	111 (56.92 %)
Epileptic seizures, *n* (%)	167 (85.64 %)
Antiepileptic drug use, *n* (%)	139 (71.28 %)
Years since diagnosis, mean (SD)	5.62 (3.66)
Functional/performance status, mean (SD)	
Karnofsky	88.11 (13.59)
Barthel	19.70 (1.24)
Order	3.89 (0.35)

*SD* standard deviation

### Agreement between patient and patient-by-proxy measurements

Table 3 summarizes the HRQOL measures for both the patients and patient-by-proxy (*n* = 246). Patients and patient-by-proxy scored similar on all scales, except for the SF-36 scale physical functioning and general health, and the QLQ-BN20 subscales visual disorder and communication deficit, and the single-item itchy skin, with patients reporting worse level of symptoms and better level of functioning than their proxies. There was also a statistically significant difference in the SF-36 PCS (−1.30; *p* = 0.02) with patients reporting a higher score than their proxies. The difference between patients and patient-by-proxy was calculated, and the proportion within ±0 (perfect agreement), 5, and 10 units was summarized with a range of 8.54 % (general health and mental health) to 76.83 % (hair loss), 19.51 % (vitality rating) to 84.55 % (hair loss), and 40.65 % (general health) to 86.59 % (role-emotional), respectively. The Bland–Altman limit of agreement (LA) for the PCS and MCS and each of the HRQOL measures revealed a fairly high agreement between the patient and patient-by-proxy rating in all HRQOL domains. The best agreement between the patient and patient-by-proxy for the SF-36 was for role-physical (points within the 95 % limit of agreement = 99.15 %, 95 % LA; −20.49–19.00) and role-emotional (99.15 %; −16.86–16.93), and for the QLQ-BN20, hair loss (98.68 %; −36.04–34.87) and bladder control (98.24 %; −43.68–38.98). A slightly poorer agreement was observed for the PCS (93.06 %; −13.63–11.03) and physical functioning (94.98 %; −31.52–25.86). Bland–Altman plots are shown for SF-36 role-physical (see Supplementary Figure S1), role-emotional (see Supplementary Figure S2), and physical functioning (see Supplementary Figure S3) to depict the extent of rater agreement across a scale range. Finally, Lin’s CCC was calculated for each HRQOL measure (Table 3). Lin’s CCC showed a moderate-to-strong relationship ranging from *r* = 0.37 (weakness in the legs) to *r* = 0.80 (physical functioning), with 79 % of the measurements greater than 0.5 [42]. The CCC for PCS was (*r* = 0.69) and MCS was (*r* = 0.55).

**Table 3:** Agreement of patient and patient-by-proxy ratings for the SF-36 and QLQ-BN20

SF-36^a^	Mean proxy	Mean patient	Mean difference (SD)	% Points within the 95% limit of agreement (LL–UL)	% Within 0 points	% Within 5 points	% Within 10 points	CCC (95 % CI)	*p* values^b^
PCS	44.11	45.57	−1.30 (6.16)	93.06 (−13.63–11.03)	–	76.42	93.09	0.69 (0.62–0.76)	0.02
MCS	41.89	42.01	0.22 (7.68)	97.11 (−15.14–15.58)	–	65.85	87.40	0.55 (0.44–0.65)	0.83
Physical functioning	80.08	83.44	−2.83 (14.35)	94.98 (−31.52–25.86)	32.93	67.07	77.64	0.80 (0.76–0.85)	0.01
Mental health	70.21	71.83	−1.06 (18.12)	95.68 (−37.30–35.17)	8.54	45.53	63.01	0.52 (0.42–0.62)	0.55
General health	56.08	60.16	−3.47 (22.03)	97.46 (−47.53–40.60)	8.54	26.02	40.65	0.54 (0.45–0.63)	0.04
Role-physical	15.29	16.20	−0.75 (9.87)	99.15 (−20.49–19.00)	43.90	47.97	74.39	0.52 (0.43–0.61)	0.29
Bodily pain	79.16	81.13	−1.72 (22.01)	96.68 (−45.74–42.29)	41.06	44.72	51.63	0.57 (0.49–0.66)	0.34
Vitality rating	58.94	61.54	−2.53 (20.74)	95.74 (−44.01–38.98)	10.57	19.51	41.46	0.58 (0.50–0.67)	0.14
Role-emotional	19.02	18.93	0.03 (8.45)	99.15 (−16.86–16.93)	62.60	66.67	86.59	0.58 (0.50–0.66)	0.76
Social functioning	78.41	77.35	1.17 (22.08)	96.69 (−42.99–45.33)	39.43	41.06	41.06	0.56 (0.48–0.65)	0.34
*EORTC Brain Cancer module QLQ*-*BN20* ^c^									
Future uncertainty	25.52	23.47	2.11 (24.69)	95.18 (−47.28–51.49)	22.76	30.08	54.88	0.47 (0.38–0.57)	0.49
Visual disorder	11.28	14.08	−2.50 (19.22)	96.92 (−40.93–35.94)	45.93	53.66	53.66	0.50 (0.40–0.60)	0.04
Motor dysfunction	14.36	13.79	0.66 (18.05)	95.63 (−35.45–36.76)	47.56	54.47	54.88	0.56 (0.48–0.65)	0.48
Communication deficit	20.86	22.96	−2.26 (23.98)	95.67 (−50.22–45.70)	74.80	82.52	82.52	0.53 (0.44–0.62)	0.03
Headaches	26.75	24.620	1.90 (26.39)	97.37 (−50.22–54.68)	57.72	65.04	65.04	0.61 (0.53–0.69)	0.50
Seizures	18.03	18.63	0.15 (23.41)	97.79 (−46.68–46.97)	68.29	76.42	76.42	0.67 (0.60–0.74)	0.88
Drowsiness	23.79	21.72	1.60 (28.65)	97.82 (−55.71–58.91)	52.85	59.76	59.76	0.47 (0.37–0.57)	0.46
Hair loss	6.01	6.94	−0.59 (17.73)	98.68 (−36.04–34.87)	76.83	84.55	84.55	0.49 (0.39–0.59)	0.50
Itchy skin	9.05	11.67	−2.95 (20.39)	98.23 (−43.74–37.84)	71.95	80.08	80.08	0.59 (0.51–0.67)	0.04
Weakness legs	5.80	6.78	−1.19 (20.38)	98.22 (−41.94–39.57)	75.61	84.15	84.15	0.37 (0.25–0.48)	0.35
Bladder control	8.44	10.56	−2.35 (20.67)	98.24 (−43.68–38.98)	74.80	82.52	82.52	0.56 (0.47–0.65)	0.06

*PCS* Physical component summary

*MCS* Mental component summary

*CCC* Concordance correlation coefficient

^a^Higher scores indicate better health

^b^Wilcoxon signed-rank test

^c^Higher scores indicate more of the symptoms

### Impact of neurocognitive deficits on patient and patient-by-proxy agreement

**Fig. 1:**
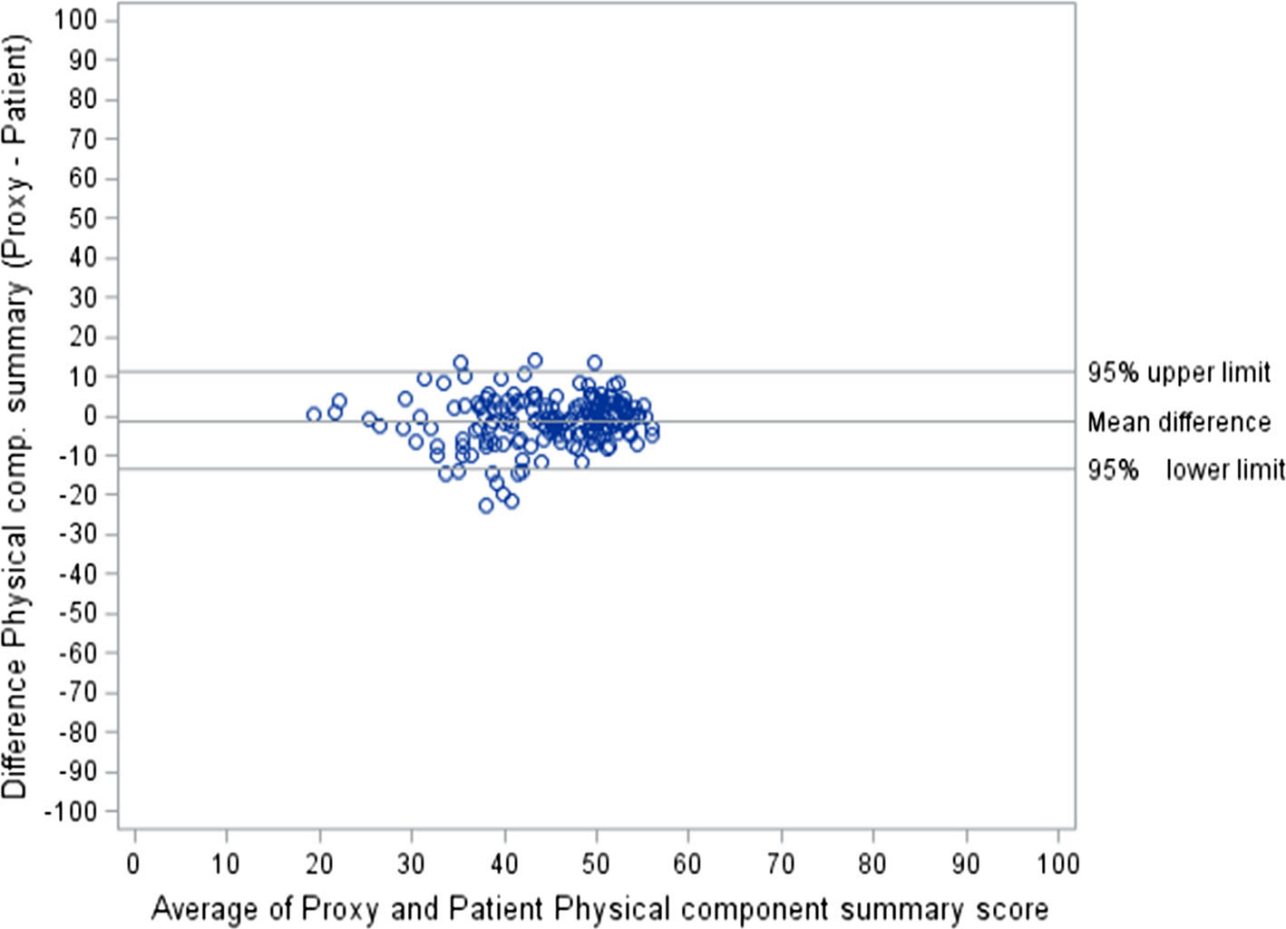
Bland–Altman plot showing the range of agreement with their 95 % limit for physical component summary

**Fig. 2:**
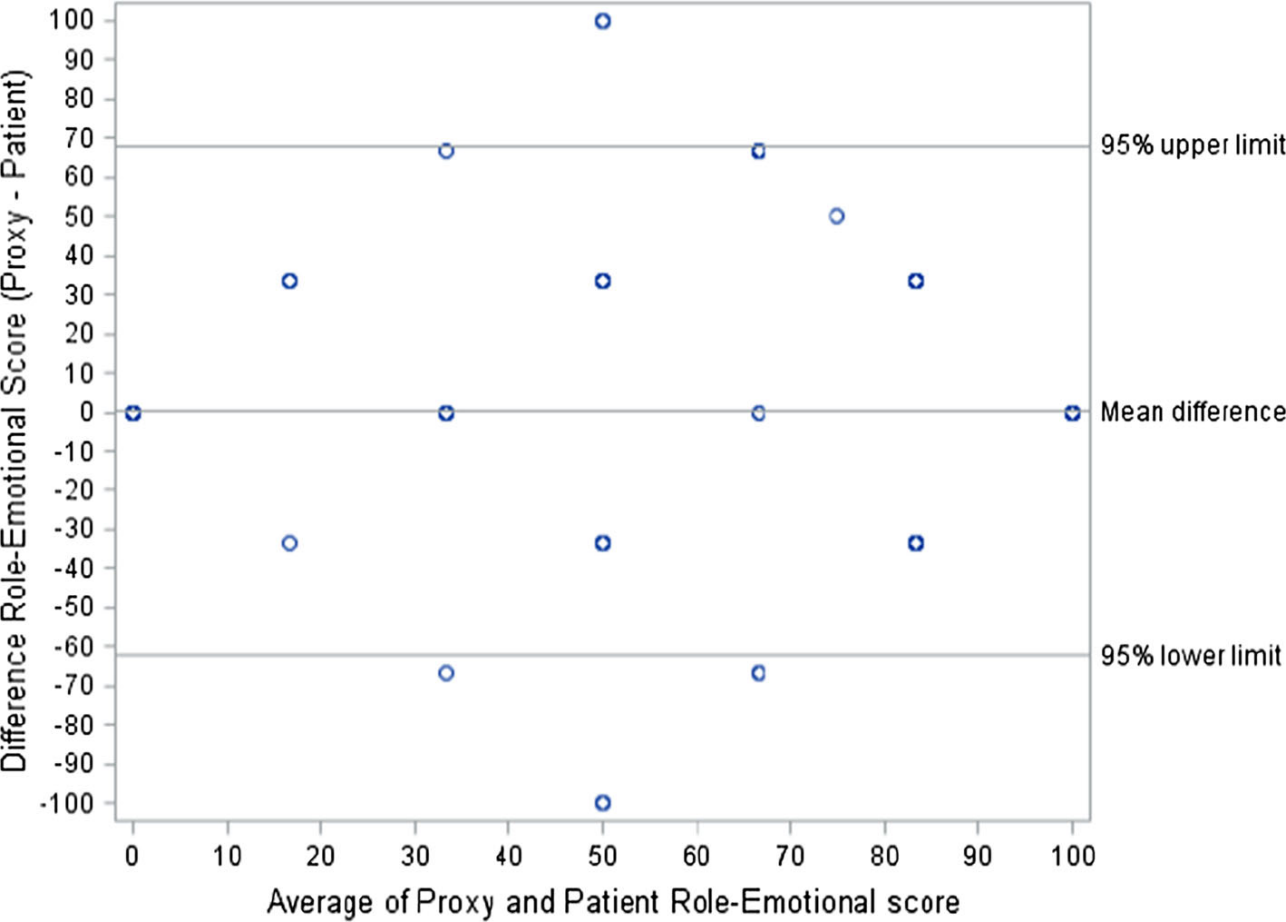
Bland–Altman plot showing the range of agreement with their 95 % limit for physical component summary

The impact of neurocognitive deficits on the agreement between patient and patient-by-proxy HRQOL scores was also examined. Out of the 195 patients who had data on neurocognitive functioning, 66 (33.85 %) patients were cognitively impaired according to our definition. The mean difference in the cognitively intact patient group was overall smaller as compared to the cognitively impaired patients (Tables 4, 5), and the Bland–Altman LA was also higher in the cognitively intact group. In the cognitively impaired group, large and statistically significant differences were observed for the QLQ-BN20 visual disorder (mean difference = −7.80; *p* = 0.001), headaches (−5.95; *p* = 0.02), itchy skin (−7.02; *p* = 0.02), and bladder control (−8.77; *p* = 0.02), indicating that cognitively impaired patients and their proxies did not agree on these scales. The difference for SF-36 physical functioning was borderline significant (−4.10; *p* = 0.05) (Table 4). As shown in Table 5, there were no statistically significant differences in any of the HRQOL scales in the cognitively intact patient group. The largest difference was observed in the QLQ-BN20 headache (5.50; *p* = 0.06) with borderline significance. The LA for PCS and MCS are shown in Figs. 1 and 2 to illustrate the extent of rater agreement across the scale range in the summarized SF-36 scales.

**Table 4:** Mean difference (patient-by-proxy minus patient) cognitively impaired patients (*n* = 66)

SF-36	Mean proxy	Mean patient	Mean difference	
			Mean (SD)	*p* values^a^
Physical component summary	40.46	42.26	−1.73 (7.10)	0.07
Mental component summary	40.52	39.75	1.41 (7.90)	0.26
Physical functioning	68.64	74.03	−4.10 (17.11)	0.05
Mental health	67.22	65.31	3.06 (17.30)	0.26
General health	47.32	51.77	−3.80 (22.69)	0.23
Role-physical	10.75	12.12	−1.29 (10.27)	0.33
Bodily pain	72.62	71.10	1.52 (21.76)	0.30
Vitality	50.38	52.34	−2.57 (20.41)	0.30
Role-emotional	15.70	15.17	0.37 (10.03)	0.63
Social functioning	71.07	68.68	2.38 (22.07)	0.23
*EORTC QLQ*-*BN20*				
Future uncertainty	29.66	31.63	−1.69 (21.42)	0.64
Visual disorder	15.44	23.95	−7.80 (18.89)	0.001
Motor dysfunction	22.60	23.08	0.10 (22.68)	0.80
Communication deficit	27.12	31.99	−4.87 (21.62)	0.08
Headaches	25.29	32.18	−5.95 (22.12)	0.02
Seizures	25.86	29.17	−1.82 (24.36)	0.38
Drowsiness	33.90	30.46	3.51 (27.95)	0.50
Hair loss	5.75	6.90	−1.19 (19.03)	0.59
Itchy skin	7.35	14.37	−7.02 (20.64)	0.02
Weakness legs	9.20	12.64	−4.76 (22.41)	0.18
Bladder control	12.43	21.26	−8.77 (24.01)	0.02

^a^Wilcoxon signed-rank test

**Table 5:** Mean difference (patient-by-proxy minus patient) cognitively intact patients (*n* = 129)

SF-36 Scores	Mean proxy	Mean patient	Mean difference	
			Mean (SD)	*p* values^a^
Physical Component Summary	47.03	47.70	−0.79 (6.19)	0.77
Mental Component Summary	43.52	43.86	−0.04 (7.52)	0.66
Physical Functioning	87.98	89.82	−1.79 (13.73)	0.32
Mental health	74.07	76.07	−1.41 (18.33)	0.52
General health	63.79	65.67	−1.44 (20.90)	0.82
Role-physical	18.42	18.52	0.09 (9.85)	0.81
Bodily pain	83.53	85.91	−2.18 (24.22)	0.36
Vitality	65.07	66.72	−1.85 (21.28)	0.53
Role-emotional	21.65	20.60	1.03 (7.06)	0.11
Social functioning	85.16	84.10	1.02 (21.54)	0.64
*EORTC QLQ*-*BN20 scores*				
Future uncertainty	22.06	19.87	2.26 (25.07)	0.67
Visual disorder	9.26	10.96	−1.71 (20.96)	0.22
Motor dysfunction	11.00	8.88	2.23 (15.87)	0.08
Communication deficit	17.78	19.84	−2.14 (25.96)	0.29
Headaches	26.60	21.13	5.50 (28.43)	0.06
Seizures	14.89	13.81	1.98 (23.49)	0.41
Drowsiness	18.77	17.26	0.98 (29.09)	0.75
Hair loss	7.05	7.81	−0.33 (17.86)	0.87
Itchy skin	9.80	9.01	0.67 (19.52)	0.72
Weakness legs	4.25	4.46	$\cong$0 (22.11)	0.99
Bladder control	6.09	5.95	−0.32 (19.52)	0.69

^a^Wilcoxon signed-rank test

## Discussion

Measuring neurocognitive functioning is essential in brain tumor patients, because this may strongly influence their HRQOL and also patient–proxy concordance levels. Although the patient is the primary source of information when measuring HRQOL, the information collected from patients with glioma may be unreliable, especially in those patients who are experiencing significant neurocognitive deterioration [14]. It is recommended to obtain proxy (i.e., caregivers) HRQOL ratings alongside a patient’s own self-report and to consider substituting patient-by-proxy ratings when a patient’s self-report is absent [43]. The original analysis of this study showed that glioma patients reported lower levels of self-reported neurocognitive functioning as measured by the MOS scales than did the healthy controls: 47.80 versus 82.40, respectively [18].

In the present study, we found that there was overall a high agreement between the patient and patient-by-proxy rating of LGG patients HRQOL in most subscales of the SF-36 and QLQ-BN20. The only statistically significant differences were observed in SF-36 physical functioning and general health and QLQ-BN20 visual disorder, communication deficit, and itchy skin. Noticeable mean differences were observed in the cognitively impaired group especially on the QLQ-BN20 scores regarding visual disorder, headaches, itchy skin, and bladder control. A noticeable difference in the cognitively intact patient group was only observed in the QLQ-BN20 headaches score. The difference between patient and patient-by-proxy ratings found in the whole group of patients could be due to the cognitively impaired patients. Although all of the differences were statistically significant, they were less than the 10 points generally accepted as clinically meaningful. However, some scores may have represented small, potentially noticeable changes in the range of 5–10 points [44] which could be important to the individual patient and warrant clinical attention.

The Bland–Altman plot revealed a high agreement between the patient and patient-by-proxy rating of HRQOL, where about 95 % of the differences between the two measurements were within the 95 % limits of agreement (prediction interval) except for SF-36 physical functioning scale and the summary component score for physical functioning (PCS). However, the limit of agreement was lower in the cognitively impaired patient group. One of the reasons for this lower agreement may be that patients, who are aware of the fact that their cognitive functions are severely affected, regard their HRQOL as poor (which is also the case in AD patients). Proxies may not fully appreciate the emotions which accompany decline of intellectual functioning.

It is important that the extent of agreement across the range of measurement be stable between the patient and patient-by-proxy [39]. Our findings showed that the agreement was poor for the central section of the scales (supplementary Figures). This was also shown by Giesinger et al. [12] who likewise stated that the possible discrepancies (i.e., ‘bias’) between the patient and patient-by-proxy are reduced by the limited range scale. The current study found a moderate-to-strong correlation between patient and patient-by-proxy scores (CCCs >0.5 for 79 % of the measurements). It is thus much easier to demonstrate agreement when a patient is experiencing either very few or many symptoms, but as the number of symptoms moves closer to 50–50 distribution, patient–proxy agreement decreases.

Regarding the methodology, our results compare quite favorably with other studies that have examined proxy ratings for general cancer patients [13, 45], brain cancer patients [12, 13], epilepsy patients [46], and stroke patients [47]. For example, a previous study in HRQOL of brain cancer patients and their proxy raters showed that intra-class correlations (ICCs) were greater than 0.5 for 77 % multi-item measures and 38 % of single-item measures [13]. However, in this previous study [13], the authors did not implement an extensive testing of patients’ neurocognitive functioning, which we did in this study. Also, the use of sound statistical techniques such as the Bland–Altman limits of agreement [16, 36–39], which are straightforward and easy to interpret, enabled us to investigate any possible relationship between the measurement error and the true value. Furthermore, our study has a large sample size and a homogeneous patient population.

Patient-by-proxy ratings may resolve compliance issues when assessing HRQOL in glioma patients with intact neurocognitive function. Probably more important in glioma patients than in any other cancer patient population, but comparable to other patients with neurological diseases associated with neurocognitive decline (e.g., Mild Cognitive Impairment and AD), patient-by-proxy ratings might also be helpful when patients cognitively deteriorate and lack the ability and insight to accurately interpret and understand the HRQOL measures. In the current study, although there was a good agreement between patient and patient-by-proxy ratings for the whole sample, there was less agreement between patient and patient-by-proxy ratings for those patients with impaired neurocognitive function compared to those patients with unimpaired neurocognitive function. While patient and patient-by-proxy ratings in such situations should not be regarded a priori as incorrect [46], insight is needed into the sources of variation between patient and proxy ratings. In a small study that compared HRQOL ratings from proxies and patients with mild AD, mild cognitive impairment, and elderly controls, it was found that overall patient–proxy agreement did not differ significantly between groups despite evident differences in neurocognitive functioning [48]. In a related study, patients with early AD generally reported a *higher* HRQOL than their proxies, and discrepancies in patient–proxy ratings were associated with the presence of anosognosia [49]. Although in the current study self-awareness was not evaluated, we found that LGG patients with cognitive deficits tended to report *more* tumor- and treatment-related symptoms and thus a *lower* HRQOL. This might indicate that potentially reduced self-awareness can be associated with both higher and lower patient HRQOL ratings relative to proxy ratings. There is currently no consensus on the best way to deal with inconsistent patient–proxy reports. While most methods rely on proxy report as a ‘gold standard’ with patient–proxy concordance taken as an indirect measure of patient (lack of) insight, the accuracy of proxy reports bares critical examination when the proxy is the caregiver. While the proxy-related factors affecting patient–proxy discrepancies are largely unknown in brain tumor patients, studies in patients with mild cognitive impairment as a prodromal phase of AD, for instance, have shown that caregivers’ cognitive skills and educational level are significant predictors of the discrepancies between caregiver ADL reports and directly assessed patient performance on ADL [50]. Furthermore, caregivers’ age, financial situation and valuation of life as a whole [51], the type of caregiver, the perspective used [52], caregiver burden [53], level of depression and anxiety [54], and caregiver health may influence the accuracy of the caregiver report. As stated earlier, patient-related factors that might affect concordance between glioma patient and proxy ratings include compromised mood and decreased neurocognitive functioning [13, 14]. Interestingly, a study that focused on screening for major depressive disorder in glioma patients [55] did not find patient–proxy agreement to be associated with severity of patient cognitive impairment, although there was frequent disagreement between glioma patients and proxies reports of depressive symptoms. A study that focused on the effect of neurocognitive functioning and performance status (KPS) on patient–proxy concordance [56] found patients and proxies to have highly congruent assessments of symptom severity regardless of patients’ neurocognitive functioning and performance status. Use of proxies as a substitute for the patient self-report of HRQOL should thus be treated with caution, always taking into consideration the possibility of potential bias.

A limitation of our study is the cross-sectional nature of the data as opposed to longitudinal data or follow-up data and generally mild neurocognitive problems in LGG patients which did not allow the detection of small mean differences between patient and patient-by-proxy ratings. Follow-up data or assessment in high-grade glioma patients with probably more neurocognitive problems might have allowed the detection of small differences between patient and patient-by-proxy HRQOL ratings. The percentage of mean differences (equal or below 0, 5, and 10 points) could be impacted by the number of possible scores on a scale [12]. A very low number of possible scores or a very large distance between two possible scores (i.e., >10 points) could distort the agreement accuracy. Also, since patients in this study had stable disease with mild neurocognitive impairment, stable LGG are not representative of the general brain tumor patient population. Investigating agreement on high-grade glioma (HGG) patients with severe neurocognitive impairment would provide additional information to assess agreement between patient and patient-by-proxy ratings. The present study specifically addressed HRQOL and did not include estimates of mood or depression. Theoretically, mood might have affected our outcomes to a certain extent as a study among patients with major depression showed that responses to self-report questionnaires are influenced by the presence of depression [57].

One issue of potential concern is basing the analyses on 195 patients from the original sample of 281 patients might result in bias. This might have been the case if patients who did not participate in neurocognitive testing were excluded, for instance, because of poor neurological or physical status. We would argue, however, that it is unlikely that bias was introduced in our study. At the time that we conducted the original study, our expectation was that only approximately 100 LGG patients would be alive in the Netherlands and meet our eligibility criteria. In fact, we were ultimately successful in identifying 281 eligible patients. Because of limited financial and personnel resources, we were only able to assess neurocognitive functioning and HRQOL in 195 consecutive patients; we assessed HRQOL only in the remaining 86 patients. There was no evidence to suggest that those who underwent both neurocognitive testing and completed HRQOL assessment differed in any significant way from those who only completed the questionnaires.

In conclusion, our data demonstrate that there is an overall high level of agreement between patient and patient-by-proxy ratings of LGG patients’ HRQOL, although the agreement for some measures is weaker in those cases where patients have neurocognitive impairment. This implies that in general, patient-by-proxy-reported outcomes can be used to replace missing patient-reported outcomes to solve compliance issues in clinical trials in this patient population. This is not the case, however, for patients with cognitive deficits who are no longer able or willing to provide self-reported data. Specifically, regarding the lack of a ‘gold standard,’ discordant patient–proxy reports should currently be considered as a parallel source of information on patient functioning. Since it is not always possible to predict which patients will suffer from progressive neurocognitive deficits, or when, it is advisable to build proxy assessments into study designs from the start of brain tumor clinical trials as is currently the case in EORTC studies 26101 (NCT01290939) and 26091 (NCT01164189).

## Electronic supplementary material

Below is the link to the electronic supplementary material.

Supplementary material 1 (DOCX 80 kb)
