# Supplementary material for: Impact of neurocognitive deficits on patient–proxy agreement regarding health-related quality of life in low-grade glioma patients
Source: Qual Life Res. 2016 Oct 15;26(4):869–80. doi: 10.1007/s11136-016-1426-z (PMC5334398; doi:10.1007/s11136-016-1426-z)
Supplement: Supplementary file 1 — Supplementary material 1 (DOCX 80 kb) [file 11136_2016_1426_MOESM1_ESM.docx]

**Supplementary Figures**


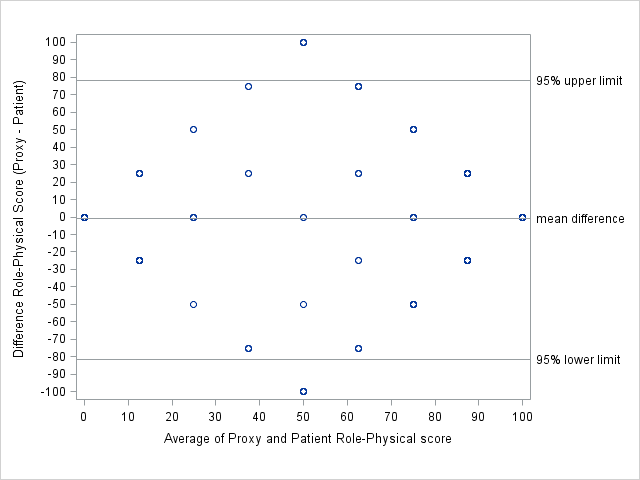


**S1: Bland–Altman plot showing the range of agreement with their 95% limit for SF-36 role-physical.**


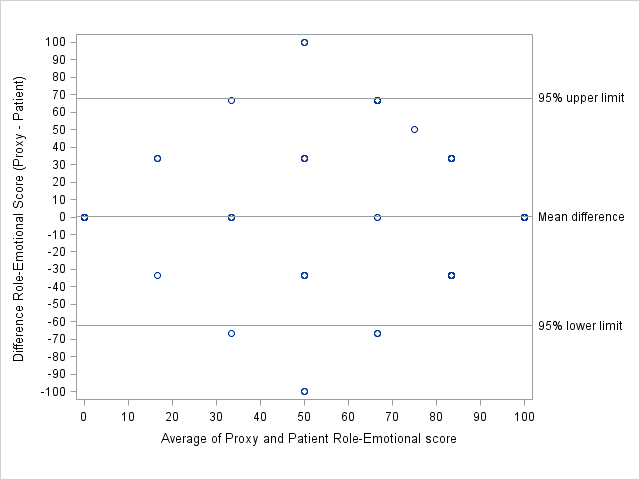


**S2: Bland–Altman plot showing the range of agreement with their 95% limit for SF-36 role-emotional.**


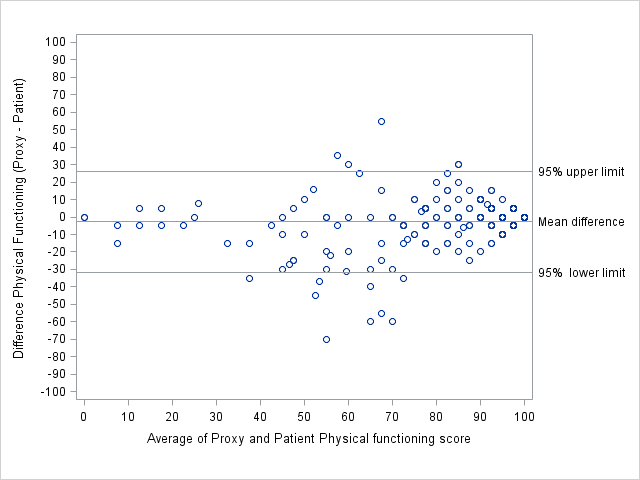


**S3: Bland–Altman plot showing the range of agreement for SF-36 physical functioning.**
